# Atlas‐Based Mapping of Traditional Chinese Medicine Effects on Tumor Microcirculation Regulation

**DOI:** 10.1002/iid3.70347

**Published:** 2026-02-04

**Authors:** I Ho, Yijie Xie, Zhipeng Liu, Dingjun Cai

**Affiliations:** ^1^ Acupuncture and Tuina School Chengdu University of Traditional Chinese Medicine Chengdu Sichuan China; ^2^ Key Laboratory of Acupuncture for Senile Disease Chengdu University of TCM, Ministry of Education/Acupuncture and Chronobiology Key Laboratory of Sichuan Province Chengdu Sichuan China

**Keywords:** angiogenesis, bibliometric analysis, immune modulation, traditional chinese medicine, tumor microcirculation

## Abstract

**Background:**

Tumor microcirculation plays a central role in the onset and progression of hypoxia, therapeutic resistance, and immune evasion within the tumor microenvironment. Traditional Chinese Medicine (TCM), characterized by its multi‐targeted and systemic regulatory properties, has garnered increasing attention for its potential to modulate this complex milieu.

**Methods:**

We systematically retrieved core literature published between 2003 and 2025 and conducted a comprehensive bibliometric and knowledge‐map analysis of 300 representative publications using CiteSpace and VOSviewer. This approach enabled the identification of key modulatory factors, underlying mechanisms, and evolving research trajectories related to TCM‐mediated regulation of tumor microcirculation.

**Results:**

Our findings reveal that TCM formulations and their active constituents—such as Tao Hong Si Wu Decoction, ginsenoside Rg3, and tanshinone IIA—modulate angiogenesis and enhance immune cell infiltration through signaling pathways including VEGF, PI3K/AKT, and HIF‐1α. Additionally, non‐pharmacological interventions such as acupuncture, electroacupuncture, and moxibustion have been shown to normalize vascular structure, modulate glycolytic activity, and reshape immune polarization. Emerging methodologies such as network pharmacology and molecular docking are increasingly utilized to unravel the complex mechanisms of TCM, facilitating the integration of traditional theories with modern scientific frameworks.

**Conclusion:**

TCM exhibits a remarkable capacity for multidimensional regulation of tumor microcirculation. Future efforts should focus on rigorous experimental validation and systems‐level modeling to accelerate its clinical translation and incorporation into integrative cancer therapy.

## 1 Introduction

The onset and progression of tumors are not solely dependent on the genetic alterations of cancer cells, but are profoundly rooted in the tumor microenvironment (TME), particularly within the aberrant tumor microcirculatory system [1, 2]. Dysregulation of tumor microcirculation results in the establishment of a hypoxic, acidic, and high‐pressure environment, which not only promotes tumor survival, invasion, and metastasis but also significantly impairs the efficacy of conventional therapies, including chemotherapy, radiotherapy, and immunotherapy [3, 4]. This presents a major challenge in cancer treatment. As global cancer incidence and mortality rates continue to rise, research on the tumor microenvironment—especially the regulation of microcirculation—has emerged as a critical frontier in the field of cancer prevention and therapy [5]. At the mechanistic level, tumor‐associated endothelial cells exhibit significant heterogeneity and functional remodeling, contributing to the formation of immune barriers that hinder the effective recognition and elimination of tumors by immune cells [6, 7]. Moreover, these endothelial cells demonstrate dependency on pro‐angiogenic factors, such as VEGF, leading to abnormal vascular structures and impaired perfusion efficiency, thus creating a systemic immunosuppressive microenvironment [8, 9]. In recent years, the introduction of the vascular normalization theory has provided novel insights for improving the tumor microenvironment and enhancing therapeutic responses [10, 11]. Despite the success of modern anti‐angiogenic therapies, their narrow therapeutic window, significant side effects, and rapid development of resistance limit their widespread application [12].

Against this backdrop, Traditional Chinese Medicine (TCM) has demonstrated significant potential in regulating tumor microcirculation due to its unique advantages in multi‐target, multi‐pathway, and holistic modulation [13, 14, 15, 16]. Studies have shown that TCM formulations and their active constituents—such as the Compound Kushen Injection, ginsenoside Rg3, and tanshinone IIA—can synergistically modulate key signaling pathways, including VEGF, Ang‐2, and, to promote vascular remodeling, enhance perfusion conditions, and increase immune cell infiltration and activation, thereby significantly boosting anti‐tumor immune responses [15, 17, 18, 19]. Furthermore, traditional non‐pharmacological TCM therapies, such as acupuncture and electroacupuncture, improve local blood flow and modulate the immune microenvironment by regulating the neuro‐immune‐endocrine network, offering valuable adjuncts to cancer treatment [20, 21]. This aligns with the TCM philosophy of “holistic diagnosis and treatment” and provides novel approaches for modern cancer therapies.

From a societal perspective, the rising incidence of cancer, driven by global aging and changes in lifestyle, continues to place a heavy burden on public health systems [22]. Patients' increasing demand for more effective, safer, and individualized treatment options with holistic regulatory capabilities is becoming ever more pronounced. TCM, with its unique multi‐target intervention and modulation capabilities, holds promise in addressing the shortcomings of modern medicine in regulating the tumor microenvironment, thereby improving therapeutic efficacy and reducing side effects [23]. This presents broad potential for clinical application. Moreover, the integration and promotion of TCM contribute to the international dissemination of Chinese culture and the diversification of global medicine, fostering a more inclusive and diverse global health system. Currently, the application of TCM in tumor microcirculation regulation faces numerous challenges [24]. First, there is a lack of a systematic and dynamic evaluation system for microcirculation, hindering the objective quantification of therapeutic efficacy and in‐depth exploration of underlying mechanisms. Second, most research remains focused on single‐pathway or single‐factor investigations, limiting the comprehensive understanding of the complex multi‐layered regulatory networks involved. Finally, the intricate components and mechanisms of TCM formulations have not been fully elucidated through modern multi‐omics and spatial omics techniques, impeding their international scientific expression and clinical translation.

Addressing these bottlenecks through the construction of bibliometric maps using advanced tools such as CiteSpace, VOSviewer, and R‐bibliometrix packages is essential for revealing research hotspots and emerging trends, which will guide future basic research and clinical practice.

## 2 Materials and Methods

### 2.1 Data Sources

**Figure 1:**
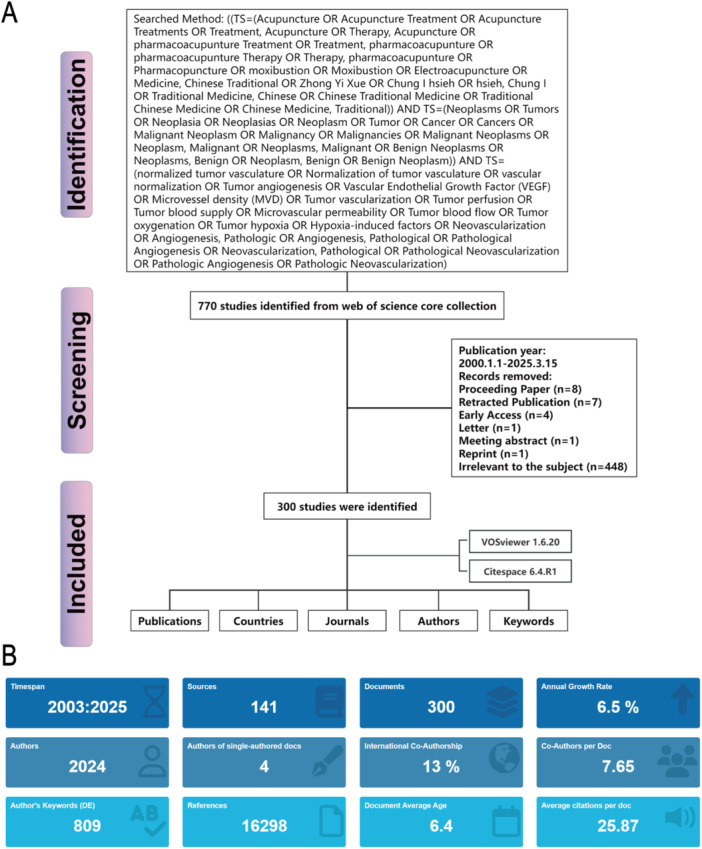
Literature screening and publication overview. (A) Flowchart illustrating the inclusion and exclusion criteria for literature screening. (B) Annual publication output on TCM‐mediated tumor microenvironment regulation from 2003 to 2025.

The data for our bibliometric analysis was sourced from the Web of Science Core Collection (WoSCC), a comprehensive and widely recognized online database. A meticulous search strategy was developed to ensure the inclusion of relevant publications (Figure 1A). The search spanned from 2003‐01‐01 to 2025‐03‐01, and focused solely on English‐language articles and reviews to standardize the language for the subsequent analysis. Publications related to TCM and tumor diseases were prioritized. Initially, 770 articles were retrieved from the database, but following a thorough review of their abstracts, 470 irrelevant publications were excluded based on their lack of relevance to the subject of TCM's role in tumor treatment (Figure 1A). Ultimately, 300 references remained for detailed analysis.

### 2.2 Analytical Methods

The search results were exported in both plain text and CSV formats for further analysis. To provide a detailed, information‐rich visualization of the research landscape surrounding TCM and tumor diseases [25], CiteSpace (version 6.4.R1) was employed for network‐based analysis. This tool allowed us to explore multiple dimensions of the research, including the relationship between institutions, authors, key references, and keywords. Methods such as co‐authorship networks, co‐citation analysis, clustering, timelines, burst detection, and dual‐map overlays were used to track trends and uncover significant patterns in the field. Additionally, to ensure a more intuitive and spatial representation of publication relationships and geographical trends, open‐source software VOSviewer (version 1.6.20) and the R‐bibliometrix package were used. These tools facilitated the analysis of research collaboration patterns, citation trends, and keyword evolution over time [26]. In this study, the selection and analysis of high‐frequency keywords were based on specific criteria. Keywords were included in the analysis only if their frequency of occurrence in the data set from 2003 to 2025 exceeded the established threshold of 13 occurrences. Broad or nonspecific terms (e.g., “research,” “study,” “data”) were excluded to ensure that only domain‐specific keywords reflecting significant research trends were retained. The clustering algorithm employed is based on the modularity method, which groups keywords based on the strength of their co‐occurrence relationships. The clustering process utilized the following parameters: ① Resolution: Set at 1 to control the granularity of the clusters, ensuring an optimal balance between over‐clustering and under‐clustering. ② Minimum cluster size: Defined as 5 to avoid forcing rare or irrelevant terms into the same cluster. ③ Cosine similarity: This metric was used to calculate the relationship between keywords based on their co‐occurrence within the same documents.

Importantly, this bibliometric analysis was not designed merely to identify high‐frequency keywords or citation patterns, but to systematically construct an integrative analytical framework for understanding how Traditional Chinese Medicine exerts coordinated regulatory effects on tumor microcirculation. By mapping co‐occurrence structures, temporal evolution, and co‐citation relationships, we aimed to translate dispersed bibliometric signals into biologically interpretable regulatory dimensions, including vascular remodeling, immune modulation, and metabolic regulation within the tumor microenvironment.

### 2.3 Data Preprocessing and Cleaning

Prior to analysis, the raw data were preprocessed to ensure its accuracy and consistency. Duplicates were removed, and only high‐quality publications from peer‐reviewed journals were retained. Keywords were standardized to reduce inconsistency in terminology (e.g., synonyms or alternate spellings). A bibliometric network was constructed by extracting and analyzing publication metadata, such as author names, institutional affiliations, citation counts, and keywords. For co‐citation and co‐authorship analysis, publications were mapped to identify clusters of research activity, and temporal trends were examined to highlight shifts in research focus.

### 2.4 Visualization and Interpretation

The visual analysis was performed with CiteSpace, which generated networks and citation patterns, allowing for a deeper understanding of the co‐citation dynamics between TCM and tumor research. Temporal clusters were identified, indicating emerging and declining research themes over the study period. To complement the CiteSpace analysis, VOSviewer was used to produce keyword co‐occurrence maps, which visually represented the concentration of research topics and how they related to each other spatially. The integration of these tools provided a comprehensive overview of research evolution, trends, and potential future directions.

In this study, visualization outputs were interpreted in the context of tumor microcirculation biology rather than as standalone descriptive maps. Specifically, keyword clusters and temporal bursts were further contextualized according to their known roles in angiogenesis, vascular normalization, immune cell trafficking, and metabolic reprogramming, thereby enabling the identification of coherent regulatory effect sets rather than isolated research trends.

## 3 Results

### 3.1 Global Overview

Our study spans a period of over 20 years, from 2003 to 2025, with an average annual growth rate of 6.5%, indicating that this field has consistently garnered academic attention and has experienced a steady increase in research activity over the past two decades (Figure 1B). A total of 300 valid publications were included in the analysis, sourced from 141 different publishing outlets, reflecting the diversity of the research content and the broad range of sources. In terms of authorship, 2024 authors contributed to the publications, with only four papers being single‐authored. On average, each article had 7.65 authors, highlighting the field's heavy reliance on collaborative research. Notably, 13% of the studies were international co‐authored works, which, though limited in number, suggest an emerging trend of cross‐national collaboration.

In terms of keywords, a total of 809 distinct author keywords were extracted, which will aid in further identifying research themes and trending directions. Regarding citations, the overall citation count reached 16,298, with an average of 25.87 citations per article, demonstrating a substantial academic impact (Figure 1B). The average publication year of the articles was 6.4 years ago, indicating that the research in this field remains relatively recent, with strong timeliness and relevance to current trends. Overall, the field has shown robust development and widespread academic engagement over the past 2 decades. Future efforts should aim to further enhance international collaborations and focus on high‐frequency keywords and emerging topics to promote sustained growth in this area.

### 3.2 Publication Trend Analysis

**Figure 2:**
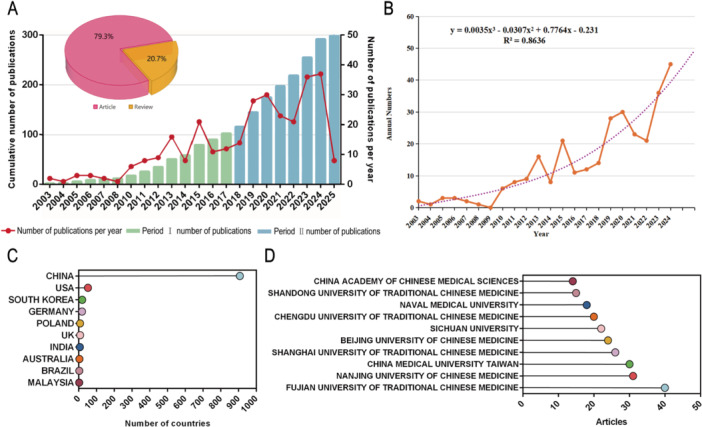
Publication trends and geographical distribution. (A) Temporal trends in annual publications related to TCM and tumor microenvironment. (B) Fitted curve representing publication growth over time. (C) Top 10 countries ranked by publication volume. (D) Top 10 institutions ranked by publication volume.

One of the most direct methods to evaluate the rise and fall of a discipline or specific research topic is to examine its annual publication trends. Based on literature data spanning from 2003 to 2025, we conducted a systematic analysis of publication trends in the field of Traditional Chinese Medicine (TCM) regulation of tumor microcirculation (Figure 2). Both the cumulative and annual numbers of publications demonstrate a steadily increasing trajectory, with a notable acceleration observed after 2014. This pattern reflects a growing research interest in recent years, suggesting that the field has entered a new phase of development post‐2014, characterized by sustained and stable growth in publication output.

In terms of publication types, original research articles dominate the landscape, accounting for 79.3% of the total publications, while review articles comprise 20.7%. This distribution indicates that although the field remains primarily driven by original investigations, reviews have also played a valuable role in synthesizing existing knowledge and guiding future inquiries (Figure 2A).

To further elucidate the evolution of research activity, we performed a polynomial regression (third‐degree) to model the annual publication trends. The resulting equation, y = 0.0035x³ − 0.0307x² + 0.7764x − 0.231, yielded a coefficient of determination of *R*² = 0.8636, indicating a strong goodness‐of‐fit and suggesting that the model effectively captures the overall growth dynamics of the field (Figure 2B). According to the model, research activity grew modestly before 2014 but experienced a marked acceleration thereafter, corroborating the observed intensification of scholarly engagement in this domain. In terms of geographic distribution, China leads overwhelmingly in the number of publications, far outpacing other nations. The United States, South Korea, and Germany follow, highlighting China's dominant position in both research input and output, while also reflecting the global diversification and increasing depth of studies in this field (Figure 2C). Among institutional contributors, the China Academy of Chinese Medical Sciences, Shandong University of Traditional Chinese Medicine, and Naval Medical University stand out for their prolific output (Figure 2D). These institutions have not only made substantial advances in basic research but have also contributed significantly to clinical translation and technological innovation, thereby propelling the field forward.

We anticipate that China will continue to play a leading role in shaping the trajectory of research in TCM and tumor microcirculation and will occupy an increasingly prominent position in the global academic landscape.

### 3.3 Overlay Map Analysis

**Figure 3:**
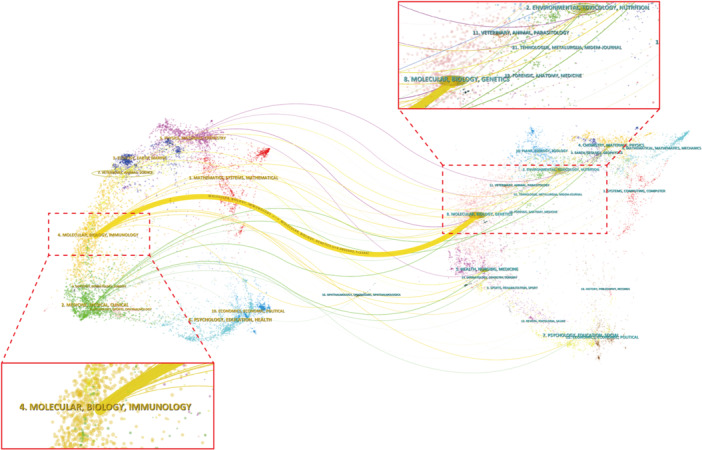
Dual‐map overlay analysis. Citation trajectories between citing journals (left) and cited journals (right), illustrating disciplinary interactions.

Overlay map analysis provides insights into the relationships and intersections between academic disciplines, unveiling the dynamic academic landscape of TCM regulation in tumor microcirculation (Figure 3). Each node in the map represents a distinct academic discipline, with connecting lines denoting the strength of relationships between them. This method helps to elucidate the intersections and interactions among various fields within the domain.

At a macro level, core life sciences such as molecular biology, genetics, forensics, anatomy, and medicine occupy central positions in the research network, maintaining close connections with environmental toxicology, nutrition, veterinary sciences, immunology, clinical medicine, psychology, and economics. The interdisciplinary interactions reflect the growing trend of integrated research, especially in the field of TCM, where the fusion of basic sciences with clinical practice is particularly pronounced. Furthermore, environmental sciences—such as ecology, earth sciences, and oceanography—are closely intertwined with biomedical research, highlighting the increasing recognition of the influence of environmental factors on the tumor microenvironment. Basic disciplines like mathematics, physics, materials science, and chemistry play crucial roles in providing theoretical support and technological methodologies. The interwoven collaborations across multiple fields not only propel advancements in biomedical sciences but also offer a more holistic perspective on clinical applications and health interventions.

### 3.4 Keyword Analysis

Keyword analysis plays a critical role in bibliometric research. A detailed and comprehensive examination of keywords uncovers emerging trends and developmental trajectories in the field of TCM for regulating tumor microcirculation, while also highlighting its potential value in addressing clinical treatment challenges. Co‐occurrence network analysis demonstrates that keywords such as “cancer,” “angiogenesis,” “expression,” and “traditional Chinese medicine” occupy central positions, forming strong associations with one another. This suggests that current research is primarily focused on key areas, including the mechanisms of tumor angiogenesis, molecular targets of TCM interventions, and the regulation of gene expression.

**Figure 4:**
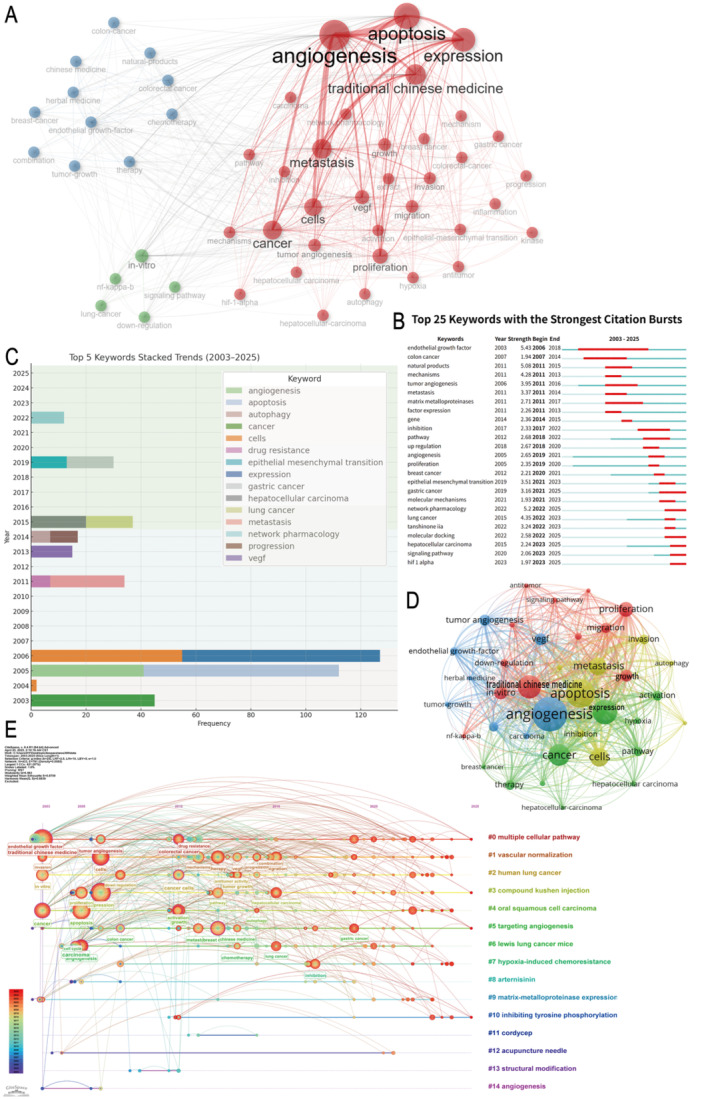
Keyword analysis. (A) Co‐occurrence network of high‐frequency keywords. (B)Top 20 keywords ranked by burst strength. (C) Top 5 Keywords Stacked Trends (2003–2025). (D) Keyword clustering visualization. (E) Timeline view of keyword evolution.

In contemporary clinical treatments, anti‐angiogenic therapies, such as bevacizumab, have become standard components in regimens for advanced solid tumors. However, the efficacy of these therapies is constrained by challenges such as vascular abnormalities, immune suppression, and drug resistance. In this context, owing to its multi‐target and low‐toxicity characteristics, TCM has gradually been integrated into intervention strategies aimed at microenvironmental regulation, particularly in optimizing vascular structure, enhancing tumor perfusion, and improving immune cell infiltration (Figure 4A).

A time‐based burst analysis of keywords further reveals the evolving dynamics of clinical focal points. Early keywords like “vascular endothelial growth factor” (VEGF) reflect concentrated research on the VEGF signaling pathway in the early 2000s. From 2010 onwards, frequent appearances of keywords such as “natural products,” “metastasis,” and “matrix metalloproteinases” underscore an increased focus on the role of natural drugs in inhibiting metastasis and remodeling the tumor microenvironment, further highlighting the growing attention to TCM in clinical support. Current clinical research has validated that certain TCM formulations (e.g., compound kushen injection, ginsenoside Rg3) can synergize with conventional therapies to improve tumor perfusion, delay tumor progression, and enhance survival rates. These advancements are gradually transitioning from laboratory research to clinical application (Figure 4B).

This study analyzes the evolution of research hotspots from 2003 to 2025, revealing dynamic shifts in the top five high‐frequency keywords and reflecting a paradigm shift within the field. During the early phase (2003–2006), the predominant keywords were largely concentrated in the realm of fundamental biology, with terms such as “apoptosis,” “angiogenesis,” and “expression” occupying central positions, reflecting a broad focus on basic cellular processes. In the intermediate phase (2007–2014), research attention gradually pivoted towards elucidating the mechanisms underlying tumor progression and therapeutic bottlenecks, as evidenced by the increasing frequency of keywords such as “metastasis,” “drug resistance,” and “autophagy.” In the recent phase (2015–2025), fueled by advancements in precision medicine and methodological innovations, terms like “hepatocellular carcinoma,” “gastric cancer,” and “epithelial‐mesenchymal transition (EMT)” have emerged, pointing to specific cancer types and their underlying mechanisms. Concurrently, the rise of “network pharmacology” marks a paradigmatic shift towards a multi‐target, big data‐driven approach in systems biology. The evolution of these top five keywords mirrors a disciplinary trajectory, transitioning from “macroscopic phenomena” to “microscopic mechanisms,” and ultimately to “systematic integration” (Figure 4C).

Further clustering and timeline analyses reveal the systematic structure and developmental trajectory of various research themes. Clusters surrounding keywords such as “VEGF” and “tumor angiogenesis” form the central axis of angiogenesis research, while terms like “expression,” “apoptosis,” and “mechanisms” focus on signaling pathways and cellular fate determination modulated by TCM. Keywords such as “traditional Chinese medicine” and “herbal treatments” reflect the expanding role of TCM interventions in clinical practice. From a temporal perspective, the prominence of terms like “vascular normalization” and “targeting angiogenesis” in recent years signals a growing emphasis on integrating microenvironmental regulation into modern tumor treatment strategies. Additionally, compounds from TCM, such as “artemisinin,” have emerged as recent research focal points, offering new insights into their anti‐angiogenic and immunomodulatory effects, thereby driving the development of novel clinical therapies (Figure 4D,E).

### 3.5 Collaboration Networks Among Countries, Institutions, and Authors

The development of any discipline or field relies heavily on collaboration and exchange among researchers. In this section, we visualize the collaborative networks between authors, countries, and institutions to reveal the multi‐layered and multi‐center cooperative structure within the field of TCM intervention in tumor microcirculation.

**Figure 5:**
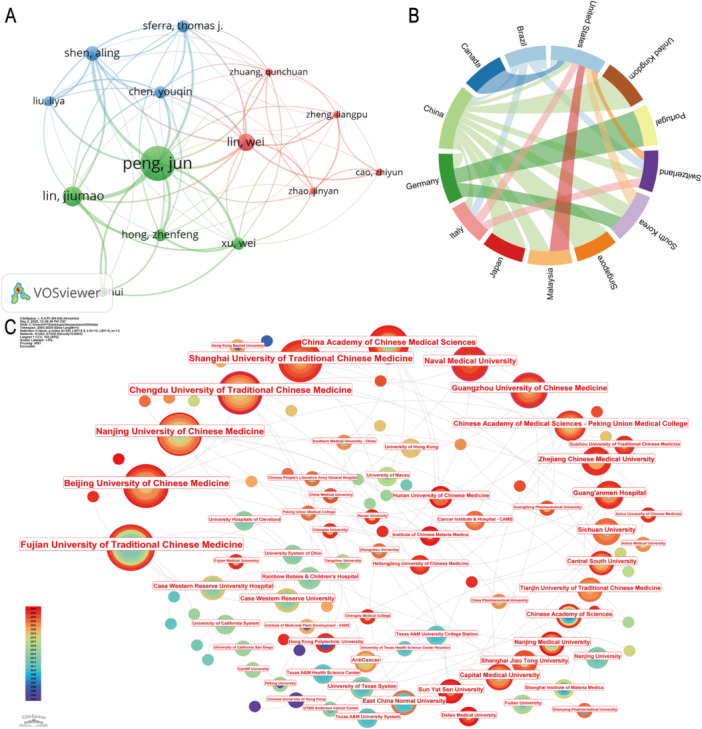
Collaboration networks among authors, countries, and institutions. (A) Author collaboration network. (B) Country collaboration network. (C) Institutional collaboration map.

At the author level, Peng Jun occupies a central position in the network, reflecting his sustained contribution and significant influence in the field. Surrounding him is a high‐frequency collaborative cluster, including key figures such as Lin Wei, Zhao Jinyan, and Cao Zhiyun, forming a stable and dynamic research community (Figure 5A). Furthermore, we employed R software to compute the academic contribution metrics for the top 10 authors globally (Table 1). The close collaboration among authors highlights the crucial role of interdisciplinary integration and team collaboration in advancing the modernization of TCM research.

**Table 1:** Top 10 authors ranked by bibliometric indicators.

Author	h_index	g_index	m_index	TC	NP	PY_start
PENG JUN	12	14	0.8	408	14	2011
LIN JIUMAO	8	8	0.533	287	8	2011
LIN WEI	7	7	0.467	186	7	2011
SHEN ALING	6	6	0.462	200	6	2013
CHEN YOUQIN	5	5	0.333	128	5	2011
HONG ZHENFENG	5	5	0.333	167	5	2011
SFERRA THOMAS J.	5	5	0.333	144	5	2011
XU WEI	5	5	0.333	162	5	2011
EFFERTH THOMAS	4	4	0.211	342	4	2007
HOFFMAN ROBERT M.	4	4	0.364	137	4	2015

At the national level, China demonstrates a prominent international leadership position in this field, with collaborative networks extending to scientific powerhouses such as the United States, Japan, and Germany. Notably, a high‐frequency research channel has been established between China and the United States. This close international cooperation not only enhances the global academic visibility of Chinese medicine within the global health system but also provides a platform for integrating advanced technologies and interdisciplinary theories into traditional medicine research. Japan and Germany are also actively engaged, further reflecting the global academic community's recognition of TCM's role in regulating tumor microenvironments and supporting therapeutic interventions (Figure 5B).

The collaborative network among institutions further affirms the positive interaction and cluster development of high‐level domestic research units. The China Academy of Chinese Medical Sciences, as the core node in the network, has established close scientific ties with institutions such as Shanghai University of Traditional Chinese Medicine, Beijing University of Chinese Medicine, Fujian University of Traditional Chinese Medicine, and Nanjing University of Chinese Medicine, driving both basic research and clinical translation in this field (Figure 5C). Overall, the collaboration networks spanning authors, institutions, and countries clearly delineate the current collaborative innovation ecosystem in TCM regulation of tumor microcirculation. This coordination has further propelled TCM toward high‐quality development and clinical translation.

### 3.6 Multiple Correspondence Analysis (MCA) and Co‐Citation Network Analysis

**Figure 6:**
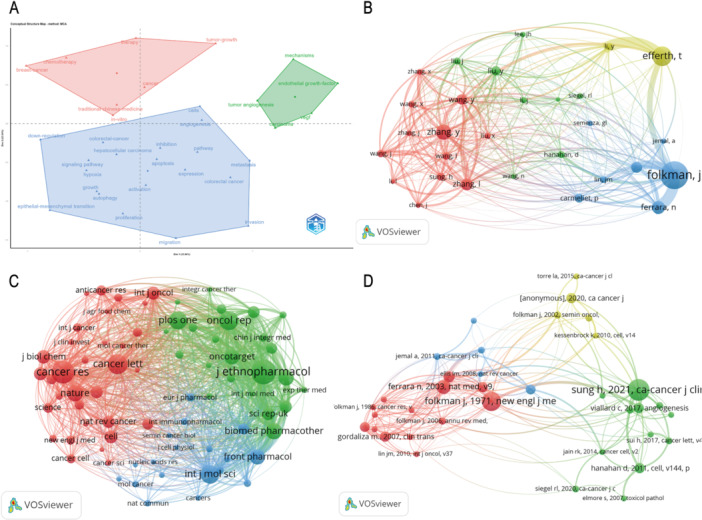
Multiple Correspondence Analysis (MCA) and co‐citation networks. (A) Multiple correspondence analysis of thematic clusters. (B) Author co‐citation network. (C) Journal co‐citation network. (D) Document co‐citation network.

Through Multiple Correspondence Analysis (MCA) and co‐citation network analysis, we gain profound insights into the relationships and interaction patterns among different concepts, authors, journals, and documents, providing valuable perspectives on research hotspots and academic development trends [27]. The MCA results, presented in a two‐dimensional coordinate system, reveal the relative distribution of research concepts and keywords, uncovering the structure and focus of the research themes. In terms of concept distribution, keywords such as “breast cancer” and “chemotherapy” occupy the upper‐left corner, indicating their high concentration and relevance in the research field. In contrast, “Traditional Chinese Medicine” and “In‐vitro” are positioned in the lower‐left corner, highlighting their unique position within the research landscape. Tumor angiogenesis (“Tumor angiogenesis”) and metastasis (“Metastasis”) are located in the upper‐right corner, signifying their prominent role in the research. Mechanistic studies and endothelial growth factor (“Endothelial growth factor”) are clustered in the lower‐right corner, further emphasizing their core role in tumor microcirculation regulation (Figure 6A).

Co‐citation network analysis further uncovers the citation and collaboration relationships between different research entities in the academic community. At the author level, works by Efferth, T., Li, Y., and Liu, X. demonstrate strong citation connections, reflecting their significant influence and academic contributions in the field (Figure 6B). Co‐citation analysis between journals shows the dominant position of top‐tier journals such as Cancer Research and International Journal of Oncology in the research domain, while also revealing close links with other journals like Oncotarget and Journal of Ethnopharmacology. This reflects the diversification and cross‐disciplinary cooperation within the field (Figure 6C). Additionally, authoritative journals such as Nature and Science maintain co‐citation relationships with numerous related journals, further validating their crucial role in knowledge dissemination and academic exchange. The co‐citation network of documents reveals the authoritative status of classic publications in academic research, with works by Folkman, J., Sung, H., and Hanahan, D. occupying the core positions of the network. This highlights their foundational contributions and historical significance in tumor microcirculation research (Figure 6D). Furthermore, we have meticulously compiled the relevant information for the top 10 most‐cited publications globally (Table 2). The mutual citation of these seminal works strengthens the theoretical foundation of the field and provides a solid academic base for future research.

**Table 2:** Top 50 most‐cited references with detailed information.

Paper	DOI	Total citations	TC per year	Normalized TC
WANG KL, 2021, BIOMED PHARMACOTHER	https://doi.org/10.1016/j.biopha.2020.111044	234	46.80	10.33
EFFERTH T, 2007, PLANTA MED	https://doi.org/10.1055/s-2007-967138	213	11.21	1.84
PANG XF, 2010, CANCER RES	https://doi.org/10.1158/0008-5472.CAN-09-3201	206	12.88	2.25
FAN TP, 2006, TRENDS PHARMACOL SCI	https://doi.org/10.1016/j.tips.2006.04.006	196	9.80	1.60
ZHAI BT, 2019, BIOMED PHARMACOTHER	https://doi.org/10.1016/j.biopha.2019.108812	191	27.29	5.26
DONG YM, 2010, CARCINOGENESIS	https://doi.org/10.1093/carcin/bgq167	150	9.38	1.64
WANG W, 2015, ACTA PHARMACOL SIN	https://doi.org/10.1038/aps.2015.24	147	13.36	4.07
EFFERTH T, 2005, DRUG RESIST UPDATE	https://doi.org/10.1016/j.drup.2005.04.003	141	6.71	1.73
LIU L, 2014, INT J CANCER	https://doi.org/10.1002/ijc.28583	124	10.33	4.07
WANG JJ, 2018, CANCER LETT	https://doi.org/10.1016/j.canlet.2017.11.037	107	13.38	3.69
YE L, 2015, ONCOL LETT	https://doi.org/10.3892/ol.2015.3459	106	9.64	2.93
MI CL, 2017, J ETHNOPHARMACOL	https://doi.org/10.1016/j.jep.2017.03.033	95	10.56	1.88
ZHANG SY, 2006, BIOL PHARM BULL	https://doi.org/10.1248/bpb.29.945	89	4.45	0.73
MARKOWITSCH SD, 2020, CANCERS	https://doi.org/10.3390/cancers12113150	86	14.33	3.50
YANCE DR, 2006, INTEGR CANCER THER	https://doi.org/10.1177/1534735405285562	83	4.15	0.68
BU L, 2020, BIOMED PHARMACOTHER	https://doi.org/10.1016/j.biopha.2020.110855	79	13.17	3.22
ZHANG XL, 2011, INT J CANCER	https://doi.org/10.1002/ijc.25909	76	5.07	1.95
WANG CL, 2019, J EXP CLIN CANC RES	https://doi.org/10.1186/s13046-019-1361-2	75	10.71	2.06
PAN Y, 2017, INT J BIOL SCI	https://doi.org/10.7150/ijbs.18969	75	8.33	1.49
YANG ZF, 2010, J CELL PHYSIOL	https://doi.org/10.1002/jcp.22261	74	4.63	0.81
ZHANG YL, 2017, CHIN MED‐UK	https://doi.org/10.1186/s13020-017-0140-2	72	8.00	1.43
GAO L, 2016, SCI REP‐UK	https://doi.org/10.1038/srep24944	72	7.20	2.16
WARTENBERG M, 2003, LAB INVEST	https://doi.org/10.1097/01.LAB.0000049348.51663.2F	72	3.13	1.48
RADOMSKA‐LESNIEWSKA DM, 2019, CENT EUR J IMMUNOL	https://doi.org/10.5114/ceji.2019.87070	71	10.14	1.95
ZHANG S, 2017, ANTICANCER RES	https://doi.org/10.21873/anticanres.11338	70	7.78	1.39
FU L, 2020, FRONT PHARMACOL	https://doi.org/10.3389/fphar.2020.00193	69	11.50	2.81
CHEN H, 2013, ONCOL REP	https://doi.org/10.3892/or.2013.2529	68	5.23	2.32
ZHU XF, 2005, MOL PHARMACOL	https://doi.org/10.1124/mol.104.009894	67	3.19	0.82
LOU CH, 2017, ONCOL REP	https://doi.org/10.3892/or.2016.5269	64	7.11	1.27
ZHAO YN, 2017, CHIN MED‐UK	https://doi.org/10.1186/s13020-017-0149-6	63	7.00	1.25
WANG XL, 2012, ONCOL REP	https://doi.org/10.3892/or.2012.1961	63	4.50	2.29
HUH JE, 2010, CANCER LETT	https://doi.org/10.1016/j.canlet.2009.11.013	63	3.94	0.69
FENG Y, 2019, MOL PHARMACEUT	https://doi.org/10.1021/acs.molpharmaceut.8b01073	62	8.86	1.71
ZUO HX, 2020, J ETHNOPHARMACOL	https://doi.org/10.1016/j.jep.2020.112835	59	9.83	2.40
LIN JM, 2013, INT J ONCOL	https://doi.org/10.3892/ijo.2012.1753	58	4.46	1.98
HSEU YC, 2011, J ETHNOPHARMACOL	https://doi.org/10.1016/j.jep.2010.11.058	56	3.73	1.44
LIN JM, 2011, MOL MED REP	https://doi.org/10.3892/mmr.2011.577	56	3.73	1.44
BAO JL, 2016, AM J CHINESE MED	https://doi.org/10.1142/S0192415X16500580	56	5.60	1.68
CHEN MX, 2016, NUTRIENTS	https://doi.org/10.3390/nu8090563	56	5.60	1.68
LI MJ, 2022, J ETHNOPHARMACOL	https://doi.org/10.1016/j.jep.2021.114689	55	13.75	4.23
DAI D, 2017, AM J CHINESE MED	https://doi.org/10.1142/S0192415X17500021	51	5.67	1.01
GUO QJ, 2015, BIOMED RES INT	https://doi.org/10.1155/2015/261620	51	4.64	1.41
JIN F, 2019, PHARM BIOL	https://doi.org/10.1080/13880209.2018.1548627	51	7.29	1.40
WANG N, 2015, J ETHNOPHARMACOL	https://doi.org/10.1016/j.jep.2015.02.025	49	4.45	1.36
ZHONG ZF, 2012, J ETHNOPHARMACOL	https://doi.org/10.1016/j.jep.2011.08.052	48	3.43	1.74
WANG KL, 2020, PHYTOMEDICINE	https://doi.org/10.1016/j.phymed.2020.153191	48	8.00	1.95
HSEU YC, 2011, J ETHNOPHARMACOL‐a	https://doi.org/10.1016/j.jep.2011.04.016	47	3.13	1.21
YANG M, 2015, ONCOL REP	https://doi.org/10.3892/or.2015.3836	47	4.27	1.30
XIE T, 2016, INT J ONCOL	https://doi.org/10.3892/ijo.2016.3416	46	4.60	1.38
PENG WJ, 2018, J ETHNOPHARMACOL	https://doi.org/10.1016/j.jep.2018.05.039	45	5.63	1.55

### 3.7 Analysis of Theme Evolution Trends

The Sankey diagram vividly illustrates the global distribution and flow of prominent research themes in the field of TCM and its regulation of tumor microcirculation across various countries and authors. The diagram highlights the dynamic evolution of these themes over time, particularly emphasizing key shifts in research focus. It underscores the centrality of themes such as “Expression,” “Apoptosis,” and “Cancer,” which are widely distributed across multiple countries, reflecting their fundamental importance within the field. In contrast, “Angiogenesis” and “Tumor angiogenesis” are predominantly concentrated in research from China and the United States, highlighting the significant contributions of these nations to the field of angiogenesis. Other terms, such as “VEGF” and “Pathway,” are also broadly represented across various regions, underscoring their pivotal role in the research landscape. In contrast, themes like “In‐vitro therapy” and “Activation” are more heavily represented in studies from Korea and Germany, demonstrating these countries' active exploration of in‐vitro therapeutic strategies and activation mechanisms.

**Figure 7:**
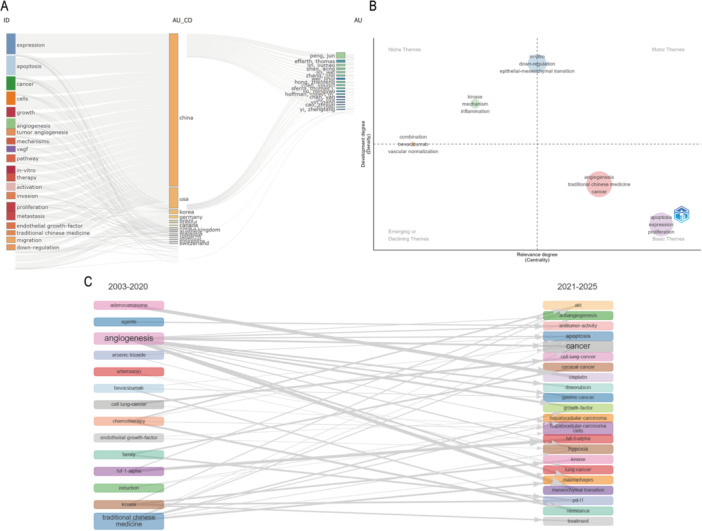
Thematic evolution analysis. (A) Sankey diagram showing topic transitions and convergence across time. (B) Centrality map illustrating the relative influence of different themes. (C) Trend chart depicting the temporal dynamics of thematic development.

The Sankey diagram also reveals the evolution of research focus, with a marked transition from “Angiogenesis” to “Vascular Normalization” (Blue), reflecting a shift in therapeutic strategies. The research focus has expanded from VEGF to a broader anti‐angiogenic therapeutic approach (Yellow), emphasizing the mainstream adoption of anti‐VEGF treatments. Furthermore, the research trajectory in TCM has shifted from a general therapeutic effect to a more refined focus on tumor microcirculation regulation (Purple), underscoring the precision of TCM mechanisms. Research on “Apoptosis” has extended into a broader exploration of “Cell Cycle Arrest” (Orange), broadening the scope of cell death mechanisms into the regulation of cell cycle processes. Additionally, there has been a transition from in‐vitro studies to in‐vivo models and clinical translation (Dark Gray), particularly in countries like Korea and Germany, indicating the gradual shift of in‐vitro research findings into clinical applications (Figure 7A).

The theme centrality map categorizes research themes based on their level of development and relevance. Foundational themes such as “Apoptosis,” “Expression,” and “Proliferation” are located in the lower‐left corner, indicating their stability and broad applicability. Emerging or declining themes, such as “Combination,” “Bevacizumab,” and “Vascular normalization,” are positioned in the central region, reflecting their fluctuating research activity over time. Core driving themes like “Angiogenesis,” “Traditional Chinese Medicine,” and “Cancer” are placed in the upper‐right corner, representing the key areas that propel the field forward. Themes such as “Kinase,” “Mechanism,” and “Inflammation” are located in the upper‐left corner, highlighting their distinctiveness and depth within specialized subfields (Figure 7B).

The theme trend graph provides further insight into the development trajectory of research themes between 2003–2020 and 2021–2025. During the period 2003–2020, “Angiogenesis” and “Artesmin” were the primary research focuses. Mid‐period research shifted towards “Chemotherapy” and “Endothelial growth factor,” while “Hypoxia‐inducible factor‐1‐alpha (HIF‐1α)” and “Kinase” emerged as key research hotspots in the later years. In the period from 2021 to 2025, “AKT” and “Anti‐tumor activity” have become central themes, reflecting their growing importance in signaling pathway regulation and anti‐tumor activity. Meanwhile, “Cancer” and “Cell lung cancer” continue to attract significant attention, indicating the sustained focus on cancer research. Additionally, themes such as “Growth factor” and “Hypoxia” show an upward trend, further highlighting the increasing importance of growth factors and hypoxic microenvironments in research (Figure 7C). By synthesizing these three types of diagrams, we have comprehensively revealed the research hotspots, development trajectory, and future trends in the regulation of tumor microcirculation by TCM, providing crucial insights for understanding the global research landscape and guiding future research directions.

## 4 Discussion

**Figure 8:**
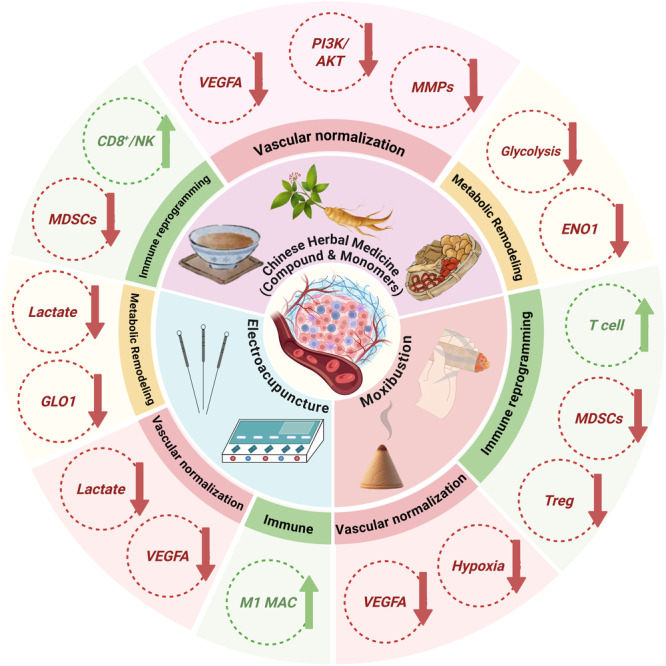
Comprehensive effects of TCM on tumor immunotherapy.

Collectively, the integrated bibliometric results indicate that the regulatory actions of Traditional Chinese Medicine on tumor microcirculation are not fragmented or pathway‐specific, but instead converge into several recurrent and interrelated regulatory effect sets that shape the tumor microenvironment in a coordinated manner. In this study, bibliometric mapping was employed to systematically delineate key research areas, core therapeutic agents, and their multidimensional regulatory mechanisms, while also identifying critical challenges and transformative directions for future development. Figure 8 provides a conceptual integration of these recurrent regulatory effect sets, illustrating how TCM interventions converge on vascular normalization, immune reprogramming, and metabolic remodeling within the tumor microenvironment.

Integrating recent advances in tumor vasculature, hypoxia biology, and immunotherapy provides a broader interpretive framework for the bibliometric patterns observed in our study, particularly the convergence of vascular modulation and immune activation [28]. From a systems‐level perspective, these regulatory effects can be broadly summarized into three interconnected sets: (1) modulation of aberrant angiogenesis toward vascular normalization, (2) improvement of microcirculatory perfusion to facilitate immune cell infiltration and reprogramming, and (3) metabolic remodeling that stabilizes the tumor microenvironment and sustains immune efficacy. Notably, these regulatory sets are not theoretical abstractions but consistently emerge from high‐frequency keywords, core clusters, and co‐citation structures identified in the bibliometric analysis. The results indicate that both TCM formulas and individual active components have emerged as central vehicles for mechanistic studies in this field. Representative compounds, such as ginsenoside Rg3, Taohong Siwu Decoction, tanshinone IIA, and Boswellia–Myrrh extracts, are frequently identified in high‐frequency keywords and core clusters, highlighting their widespread attention and considerable potential in regulating tumor angiogenesis, maintaining vascular stability, and promoting immune cell infiltration. Recent advances have highlighted vascular normalization as a central mediator linking hypoxia reduction, immune cell trafficking, and improved response to immunotherapy, forming a key mechanistic basis for interpreting these trends [29, 30]. For instance, Taohong Siwu Decoction, a classic formulation embodying the TCM principle of “invigorating blood and resolving stasis,” has been shown to modulate the aberrant vascular perfusion in breast cancer models via multiple pathways, including the downregulation of VEGF‐A, regulation of HIF‐1α expression, and inhibition of MMP9 activity, thereby enhancing chemotherapy sensitivity and immune efficacy while improving local microcirculatory structure [31]. In parallel, Xiatansan Jiedu Decoction exhibits unique clinical potential in anti‐angiogenesis by inhibiting key enzymes such as MMP‐2 and MMP‐9, reflecting the “phlegm syndrome theory” in anti‐angiogenic strategies.

On the individual component front, ginsenoside Rg3 has been extensively studied and established as a dual‐functional natural product [32]. It effectively suppresses angiogenesis via the VEGF/PI3K/AKT signaling pathway while also inducing the polarization of tumor‐associated macrophages towards the M1 phenotype, thereby enhancing their anti‐tumor properties and achieving synergistic regulation of both immune and vascular systems [33]. Multiple high‐quality reviews demonstrate that representative TCM‐derived compounds—including ginsenosides, curcumin, and astragaloside IV—modulate VEGF signaling, myeloid‐derived suppressor cells, and T‐cell function, consistent with these mechanistic clusters [34]. Additionally, active components like indirubin and myricetin intervene in vascular maturation and immune activation processes through the JAK/STAT3 and VEGFR2‐p38/MAPK pathways, respectively, demonstrating favorable effects in improving microenvironmental structure and inhibiting tumor progression [35, 36]. Tanshinone IIA also exhibits broad‐spectrum regulatory functions, modulating angiogenesis through multiple signaling axes, such as β‐catenin/VEGF, EGFR/IGF1R inhibition, and Ras/Raf/MEK/ERK and PI3K/AKT/mTOR blockade, underscoring its pivotal role in mechanistic studies [18, 37]. Recent pharmacological studies have further enriched these findings, unveiling novel mechanistic roles of natural products in immune modulation and vascular homeostasis, providing new evidence for understanding the systemic effects of TCM [38, 39, 40].

It is noteworthy that the bibliometric analysis highlights frequent keywords such as “VEGF,” “PI3K/AKT,” “HIF‐1α,” “TME,” “MMP,” and “CD8 + T cell,” suggesting a paradigm shift from traditional “single‐target anti‐angiogenesis” approaches towards the construction of an integrated “vascular–immune–metabolism” network. This shift facilitates more comprehensive and dynamic interventions within the tumor microenvironment. Moreover, the potential of natural products in regulating glycolysis and lactate metabolism has garnered attention. For instance, tanshinone IIA modulates angiogenesis by downregulating α‐enolase and AMPK phosphorylation, indirectly inhibiting HIF‐1α expression [41]. Meanwhile, Bai Zhu lactone I regulates glucose metabolism, apoptosis, and tumor stemness through the AKT/mTOR pathway, revealing a novel mechanism wherein metabolic reprogramming facilitates vascular stabilization. These findings demonstrate that TCM‐mediated microcirculatory regulation is not limited to single targets or local mechanisms, but reflects a high degree of coupling across multiple systems and pathways. Rather than acting through isolated molecular targets, these formulas and monomers exemplify how Traditional Chinese Medicine achieves coordinated regulation across vascular, immune, and metabolic dimensions, reinforcing the concept of a unified microcirculation‐centered regulatory effect set.

Beyond herbal medicine, acupuncture, a crucial non‐pharmacological aspect of TCM, has also shown increasing promise in regulating tumor microcirculation [42]. The bibliometric data reveals that acupuncture and moxibustion are gaining attention, especially for their significant effects in promoting vascular normalization, improving immune suppression, and modulating local metabolism. Recent studies have confirmed that electroacupuncture improves the metabolic state of tumor endothelial cells by downregulating glycolytic molecules like GLO1, reducing lactate accumulation, and inhibiting pathological angiogenesis [43]. This optimizes local perfusion and enhances the permeability and efficacy of anti‐tumor drugs. Additionally, electroacupuncture induces macrophage polarization to the M1 phenotype, synergistically enhancing the immune functionality of the microenvironment [44]. By regulating the expression of angiogenic factors such as VEGFA and NRP‐1, electroacupuncture further contributes to the normalization of vascular structures [45]. In combined interventions, moxibustion alongside chemotherapy agents such as cisplatin synergistically downregulates factors like HIF‐1α and VEGFA, significantly improving tumor hypoxia and promoting the infiltration of CD8 + T cells, Th1 cells, and M1 macrophages, while suppressing immunosuppressive cells like Tregs and MDSCs [46]. This dual enhancement of anti‐angiogenesis and immune activation underscores the therapeutic promise of TCM [47]. However, most evidence for acupuncture or moxibustion remains limited by small sample sizes and methodological constraints, and therefore these findings should be interpreted with caution [48]. Despite these promising findings, the bibliometric data also reveal several challenges in current research, particularly regarding the depth and methodology of studies. Many investigations remain at the level of network pharmacology and molecular docking, lacking functional validation through cellular assays, animal models, and clinical translation. This results in fragmented mechanistic understanding and uncertainty in findings. Furthermore, current mechanistic studies often focus on core pathways such as VEGF/PI3K, with limited attention to cellular functions, such as endothelial cell mitochondrial dynamics, metabolite networks, and cell‐to‐cell communication mechanisms. This narrow focus fails to fully capture the complex, multi‐target, and multi‐pathway regulatory features inherent in TCM formulations. Moreover, the holistic philosophy, dynamic regulation, and individualized treatment approach unique to TCM are often underrepresented in experimental design. Most studies simplify complex formulas to individual components, thereby weakening their multi‐component and multi‐target intervention advantages and making it difficult to accurately reflect their clinical effects.

Furthermore, future research should place greater emphasis on integrative multi‐omics approaches to explore the complexities at the mechanistic level. Single‐omics approaches often fall short in fully revealing the intricate tumor microcirculation regulatory network. A combined analysis of transcriptomics, metabolomics, and proteomics data provides higher resolution at the systems level. For instance, transcriptomics can reveal the expression profiles of key genes, while metabolomics captures tumor‐driven metabolic reprogramming in the microenvironment. By combining both, biases from single‐dimensional data can be avoided, reducing the risk of false positives [49]. Moreover, integrative multi‐omics with pathway enrichment and network analysis has been shown to significantly improve the resolution of signaling pathways, enabling researchers to more accurately pinpoint key regulatory nodes in the context of complex signaling networks [50].

In the research of tumor angiogenesis and immune microenvironment, multi‐omics strategies have not only elucidated the hierarchical regulation of classical pathways such as VEGF/PI3K/AKT, but also discovered coupling mechanisms between metabolic reprogramming and immune suppression, providing systematic evidence for the multi‐target actions of TCM formulations [51]. Furthermore, in breast cancer research, integrating transcriptomics and metabolomics has revealed associations between glycolysis‐related metabolites and immune cell infiltration, improving research accuracy and providing potential target references for TCM interventions [52]. Therefore, the integration of multi‐omics technologies into TCM research on regulating tumor microcirculation not only helps reduce false positives and enhances pathway resolution but also strengthens the reliability and clinical translation potential of results at the systems level. Future studies combining spatial omics and single‐cell multi‐omics will further advance our comprehensive understanding of the multi‐target mechanisms of TCM.

Additionally, the design of future clinical trials should expand endpoint indicators. The commonly adopted RECIST standard primarily relies on imaging to assess tumor size changes, which fails to adequately reflect improvements in the tumor microenvironment and functional alterations [53]. Therefore, in TCM intervention research, alternative or complementary endpoints more aligned with the mechanisms of TCM should be explored, such as introducing TCM‐specific quality‐of‐life scales. Studies have shown that the QLASTCM‐Ga (gastric cancer) and QLASTCM‐Lu (lung cancer) scales, developed based on TCM theory, exhibit excellent reliability, validity, and clinical sensitivity, allowing for sensitive reflection of patient syndrome differences and treatment responses [54, 55]. Meanwhile, cross‐sectional studies indicate a significant increase in the application of patient‐reported outcomes (PROs) in TCM clinical trials, with TCM symptom scales and quality‐of‐life scales gradually becoming key evaluation tools, although their standardization and internationalization still require further development [56]. These comprehensive endpoints not only more accurately assess TCM's effects on microcirculation and immune environments but also contribute to the standardization and international application of TCM interventions in clinical settings.

In summary, TCM demonstrates significant mechanistic diversity and broad intervention potential in regulating tumor microcirculation, encompassing angiogenesis, immune activation, and metabolic reprogramming. The synergistic interaction between herbal formulas, natural products, acupuncture, and other interventions forms a complementary framework, establishing a novel research paradigm centered on “vascular–immune–metabolism” coordination. Emerging evidence also indicates that natural products may enhance responses to PD‐1/PD‐L1 blockade by reducing PD‐L1, Tregs expression or increasing CD4^+^, CD8⁺ infiltration, which further contextualizes these immune‐related findings [57]. Moreover, bibliometric approaches inherently cannot evaluate methodological quality, and citation patterns may reflect field size rather than evidence strength. However, to advance the field to higher levels, it is essential to overcome current research bottlenecks centered on prediction models and transition towards system‐level studies that integrate mechanistic validation with spatial and functional omics techniques. Additionally, TCM's holistic philosophy and intervention advantages must be more thoroughly integrated into research strategies. Only by doing so can we elevate the scientific value and international discourse of TCM in cancer treatment.

## 5 Conclusion

This study systematically reviews the research on the regulation of tumor microcirculation by TCM over the past 22 years using VOSviewer and CiteSpace, revealing key hotspots and emerging trends in the field. Current research primarily focuses on how TCM, through the regulation of signaling pathways such as VEGF, PI3K/AKT, and HIF‐1α, improves vascular function and enhances immune responses. Additionally, meridian‐based therapies such as acupuncture and moxibustion contribute to the improvement of the microcirculatory environment by modulating metabolic and immune states. Network pharmacology and molecular docking approaches are widely employed for mechanistic predictions, although the lack of experimental validation remains a major bottleneck. Moving forward, it is essential to integrate multi‐omics and high‐resolution technologies to further explore the mechanisms underlying TCM intervention in the tumor microenvironment, while also advancing clinical translation.

## Author Contributions

**I Ho:** writing – original draft, writing – review and editing, software, conceptualization, data curation, formal analysis, methodology, visualization. **Dingjun Cai:** writing – review and editing.

## Conflicts of Interest

The authors declare no conflicts of interest.

## References

[1] L. Bejarano , M. J. C. Jordāo , and J. A. Joyce , “Therapeutic Targeting of the Tumor Microenvironment,” Cancer Discovery 11, no. 4 (April 2021): 933–959, 10.1158/2159-8290.CD-20-1808. 33811125

[2] A. Tiwari , R. Trivedi , and S.‐Y. Lin , “Tumor Microenvironment: Barrier or Opportunity Towards Effective Cancer Therapy,” Journal of Biomedical Science 29, no. 1 (October 2022): 83, 10.1186/s12929-022-00866-3. 36253762 PMC9575280

[3] N. Srivastava , S. S. Usmani , R. Subbarayan , R. Saini , and P. K. Pandey , “Hypoxia: Syndicating Triple Negative Breast Cancer Against Various Therapeutic Regimens,” Frontiers in Oncology 13 (2023): 1199105, 10.3389/fonc.2023.1199105. 37492478 PMC10363988

[4] J. Roesler , D. Spitzer , X. Jia , et al., “Disturbance in Cerebral Blood Microcirculation and Hypoxic‐Ischemic Microenvironment Are Associated With the Development of Brain Metastasis,” Neuro‐Oncology 26, no. 11 (November 2024): 2084–2099, 10.1093/neuonc/noae094. 38831719 PMC11534324

[5] P. Vaupel , “Tumor Microenvironmental Physiology and Its Implications for Radiation Oncology,” Seminars in Radiation Oncology 14, no. 3 (July 2004): 198–206, 10.1016/j.semradonc.2004.04.008. 15254862

[6] S. K. Calderwood , “Tumor Heterogeneity, Clonal Evolution, and Therapy Resistance: An Opportunity for Multitargeting Therapy,” Discovery Medicine 15, no. 82 (March 2013): 188–194. 23545047 PMC4083486

[7] K. E. Fathers , C. M. Stone , K. Minhas , et al., “Heterogeneity of Tie2 Expression in Tumor Microcirculation,” American Journal of Pathology 167, no. 6 (December 2005): 1753–1762, 10.1016/S0002-9440(10)61256-4. 16314485 PMC1613180

[8] C.‐L. Kuo , H. Y. Chou , H. W. Lien , et al., “A Fc‐VEGF Chimeric Fusion Enhances PD‐L1 Immunotherapy via Inducing Immune Reprogramming and Infiltration in the Immunosuppressive Tumor Microenvironment,” Cancer Immunology, Immunotherapy 72, no. 2 (February 2023): 351–369, 10.1007/s00262-022-03255-9. 35895109 PMC9870840

[9] S. A. Patel , M. B. Nilsson , X. Le , T. Cascone , R. K. Jain , and J. V. Heymach , “Molecular Mechanisms and Future Implications of VEGF/VEGFR in Cancer Therapy,” Clinical Cancer Research 29, no. 1 (January 2023): 30–39, 10.1158/1078-0432.CCR-22-1366. 35969170 PMC10274152

[10] Y. Choi and K. Jung , “Normalization of the Tumor Microenvironment by Harnessing Vascular and Immune Modulation to Achieve Enhanced Cancer Therapy,” Experimental & Molecular Medicine 55, no. 11 (November 2023): 2308–2319, 10.1038/s12276-023-01114-w. 37907742 PMC10689787

[11] C. Viallard and B. Larrivée , “Tumor Angiogenesis and Vascular Normalization: Alternative Therapeutic Targets,” Angiogenesis 20, no. 4 (November 2017): 409–426, 10.1007/s10456-017-9562-9. 28660302

[12] J. Tu , H. Liang , C. Li , et al., “The Application and Research Progress of Anti‐Angiogenesis Therapy in Tumor Immunotherapy,” Frontiers in Immunology 14 (2023): 1198972, 10.3389/fimmu.2023.1198972. 37334350 PMC10272381

[13] Y. Lu , C. Zhou , M. Zhu , et al., “Traditional Chinese Medicine Syndromes Classification Associates With Tumor Cell and Microenvironment Heterogeneity in Colorectal Cancer: A Single Cell RNA Sequencing Analysis,” Chinese Medicine 16, no. 1 (December 2021): 133, 10.1186/s13020-021-00547-7. 34876190 PMC8650518

[14] L. Xia , X. Liu , W. Mao , et al., “Panax Notoginseng Saponins Normalises Tumour Blood Vessels by Inhibiting EphA2 Gene Expression to Modulate the Tumour Microenvironment of Breast Cancer,” Phytomedicine 114 (2023): 154787, 10.1016/j.phymed.2023.154787. 37060724

[15] J. Zhang , J. Gao , J. Cui , et al., “Tumor‐Associated Macrophages in Tumor Progression and the Role of Traditional Chinese Medicine in Regulating TAMs to Enhance Antitumor Effects,” Frontiers in Immunology 13 (2022): 1026898, 10.3389/fimmu.2022.1026898. 36311793 PMC9611775

[16] Y. Zhang , Y. Lou , J. Wang , C. Yu , and W. Shen , “Research Status and Molecular Mechanism of the Traditional Chinese Medicine and Antitumor Therapy Combined Strategy Based on Tumor Microenvironment,” Frontiers in Immunology 11 (2021): 609705, 10.3389/fimmu.2020.609705. 33552068 PMC7859437

[17] N. Luo , K. Zhang , X. Li , Y. Hu , and L. Guo , “Tanshinone IIA Destabilizes SLC7A11 by Regulating PIAS4‐Mediated SUMOylation of SLC7A11 Through KDM1A, and Promotes Ferroptosis in Breast Cancer,” Journal of Advanced Research 69 (March 2025): 313–327, 10.1016/j.jare.2024.04.009. 38615741 PMC11954794

[18] H. Sui , J. Zhao , L. Zhou , et al., “Tanshinone IIA Inhibits β‐Catenin/VEGF‐Mediated Angiogenesis by Targeting TGF‐β1 in Normoxic and HIF‐1α in Hypoxic Microenvironments in Human Colorectal Cancer,” Cancer Letters 403 (September 2017): 86–97, 10.1016/j.canlet.2017.05.013. 28602978

[19] Y. Zhu , A. Wang , S. Zhang , et al., “Paclitaxel‐Loaded Ginsenoside Rg3 Liposomes for Drug‐Resistant Cancer Therapy by Dual Targeting of the Tumor Microenvironment and Cancer Cells,” Journal of Advanced Research 49 (July 2023): 159–173, 10.1016/j.jare.2022.09.007. 36167294 PMC10334248

[20] N. Li , Y. Guo , Y. Gong , et al., “The Anti‐Inflammatory Actions and Mechanisms of Acupuncture From Acupoint to Target Organs via Neuro‐Immune Regulation,” Journal of Inflammation Research 14 (2021): 7191–7224, 10.2147/JIR.S341581. 34992414 PMC8710088

[21] N. Wang , L. Zhao , D. Zhang , and F. Kong , “Research Progress on the Immunomodulatory Mechanism of Acupuncture in Tumor Immune Microenvironment,” Frontiers in Immunology 14 (2023): 1092402, 10.3389/fimmu.2023.1092402. 36865562 PMC9971227

[22] L. Partridge , J. Deelen , and P. E. Slagboom , “Facing Up to the Global Challenges of Ageing,” Nature 561, no. 7721 (September 2018): 45–56, 10.1038/s41586-018-0457-8. 30185958

[23] S. Wang , S. Long , Z. Deng , and W. Wu , “Positive Role of Chinese Herbal Medicine in Cancer Immune Regulation,” American Journal of Chinese Medicine 48, no. 7 (2020): 1577–1592, 10.1142/S0192415X20500780. 33202152

[24] Y. Li , Y. Wang , W. Tai , et al., “Challenges and Solutions of Pharmacokinetics for Efficacy and Safety of Traditional Chinese Medicine,” Current Drug Metabolism 16, no. 9 (2015): 756–776, 10.2174/138920021609151201114223. 26630907

[25] M. Sabe , T. Pillinger , S. Kaiser , et al., “Half a Century of Research on Antipsychotics and Schizophrenia: A Scientometric Study of Hotspots, Nodes, Bursts, and Trends,” Neuroscience & Biobehavioral Reviews 136 (2022): 104608, 10.1016/j.neubiorev.2022.104608. 35303594

[26] H. Arruda , E. R. Silva , M. Lessa , D. Proença, Jr. , and R. Bartholo , “VOSviewer and Bibliometrix,” Journal of the Medical Library Association 110, no. 3 (July 2022): 392–395, 10.5195/jmla.2022.1434. 36589296 PMC9782747

[27] D. Florensa , P. Godoy , J. Mateo , et al., “The Use of Multiple Correspondence Analysis to Explore Associations Between Categories of Qualitative Variables and Cancer Incidence,” IEEE Journal of Biomedical and Health Informatics 25, no. 9 (September 2021): 3659–3667, 10.1109/JBHI.2021.3073605. 33857006

[28] P. Sidaway , “T‐DXd Is Effective After T‐DM1,” Nature Reviews Clinical Oncology 20, no. 7 (July 2023): 426, 10.1038/s41571-023-00779-6. 37173585

[29] K. Miao , W. Liu , J. Xu , Z. Qian , and Q. Zhang , “Harnessing the Power of Traditional Chinese Medicine Monomers and Compound Prescriptions to Boost Cancer Immunotherapy,” Frontiers in Immunology 14 (2023): 1277243, 10.3389/fimmu.2023.1277243. 38035069 PMC10684919

[30] S. Kakkad , B. Krishnamachary , D. Jacob , et al., “Molecular and Functional Imaging Insights Into the Role of Hypoxia in Cancer Aggression,” Cancer and Metastasis Reviews 38, no. 1–2 (June 2019): 51–64, 10.1007/s10555-019-09788-3. 30840168 PMC6625878

[31] H. Jiang , M. Li , K. Du , et al., “Traditional Chinese Medicine for Adjuvant Treatment of Breast Cancer: Taohong Siwu Decoction,” Chinese Medicine 16, no. 1 (December 2021): 129, 10.1186/s13020-021-00539-7. 34857023 PMC8638166

[32] M. Nakhjavani , E. Smith , A. R. Townsend , T. J. Price , and J. E. Hardingham , “Anti‐Angiogenic Properties of Ginsenoside Rg3,” Molecules 25, no. 21 (October 2020): 4905, 10.3390/molecules25214905. 33113992 PMC7660320

[33] D. Zeng , J. Wang , P. Kong , C. Chang , J. Li , and J. Li , “Ginsenoside Rg3 Inhibits HIF‐1α and VEGF Expression in Patient With Acute Leukemia via Inhibiting the Activation of PI3K/Akt and ERK1/2 Pathways,” International Journal of Clinical and Experimental Pathology 7, no. 5 (2014): 2172–2178. 24966925 PMC4069960

[34] Y. F. Huang , C. Bai , F. He , Y. Xie , and H. Zhou , “Review on the Potential Action Mechanisms of Chinese Medicines in Treating Coronavirus Disease 2019 (COVID‐19),” Pharmacological Research 158 (2020): 104939, 10.1016/j.phrs.2020.104939. 32445956 PMC7239792

[35] J. Lai , Yh Liu , C. Liu , et al., “Indirubin Inhibits LPS‐Induced Inflammation via TLR4 Abrogation Mediated by the NF‐kB and MAPK Signaling Pathways,” Inflammation 40, no. 1 (February 2017): 1–12, 10.1007/s10753-016-0447-7. 27718095

[36] X. Zhang , Y. Song , Y. Wu , et al., “Indirubin Inhibits Tumor Growth by Antitumor Angiogenesis via Blocking VEGFR2‐mediated JAK/STAT3 Signaling in Endothelial Cell,” International Journal of Cancer 129, no. 10 (November 2011): 2502–2511, 10.1002/ijc.25909. 21207415

[37] N. Y. Kim , Y. Y. Jung , M. H. Yang , et al., “Tanshinone IIA Exerts Autophagic Cell Death Through Down‐Regulation of β‐Catenin in Renal Cell Carcinoma Cells,” Biochimie 200 (September 2022): 119–130, 10.1016/j.biochi.2022.05.018. 35654241

[38] S. Tian , X. Liao , W. Cao , et al., “GSFM: A Genome‐Scale Functional Module Transformation to Represent Drug Efficacy for in Silico Drug Discovery,” Acta Pharmaceutica Sinica B 15, no. 1 (January 2025): 133–150, 10.1016/j.apsb.2024.08.017. 40041913 PMC11873659

[39] S. Tian , Y. Li , J. Xu , et al., “COIMMR: A Computational Framework to Reveal the Contribution of Herbal Ingredients Against Human Cancer via Immune Microenvironment and Metabolic Reprogramming,” Briefings in Bioinformatics 24, no. 6 (2023): 1–14, https://academic.oup.com/bib/article/24/6/bbad346/7294469. 10.1093/bib/bbad346 PMC10564268 37816138

[40] S. Tian , J. Zhang , S. Yuan , et al., “Exploring Pharmacological Active Ingredients of Traditional Chinese Medicine by Pharmacotranscriptomic Map in ITCM,” Briefings in Bioinformatics 24, no. 2 (2023): 1–14, https://academic.oup.com/bib/article/24/2/bbad027/7017365. 10.1093/bib/bbad027 36719094

[41] T. H. Son , S. H. Kim , H. L. Shin , et al., “3‐Hydroxytanshinone Inhibits the Activity of Hypoxia‐Inducible Factor 1‐α by Interfering With the Function of α‐Enolase in the Glycolytic Pathway,” Molecules 29, no. 10 (May 2024): 2218, 10.3390/molecules29102218. 38792080 PMC11123766

[42] M. Wang , W. Liu , J. Ge , and S. Liu , “The Immunomodulatory Mechanisms for Acupuncture Practice,” Frontiers in Immunology 14 (2023): 1147718, 10.3389/fimmu.2023.1147718. 37090714 PMC10117649

[43] Y.‐X. Wan , X. W. Qi , Y. Y. Lian , et al., “Electroacupuncture Facilitates Vascular Normalization by Inhibiting Glyoxalase1 in Endothelial Cells to Attenuate Glycolysis and Angiogenesis in Triple‐Negative Breast Cancer,” Cancer Letters 598 (2024): 217094, 10.1016/j.canlet.2024.217094. 38945204

[44] X. Qi , Y. Lian , Z. Fan , et al., “Electroacupuncture Normalized Tumor Vasculature by Downregulating glyoxalase‐1 to Polarize Tumor‐Associated Macrophage to M1 Phenotype in Triple‐Negative Breast Cancer,” International Immunopharmacology 147 (2025): 113988, 10.1016/j.intimp.2024.113988. 39778275

[45] M. Dai , K. Qian , Q. Ye , et al., “Specific Mode Electroacupuncture Stimulation Mediates the Delivery of NGF Across the Hippocampus Blood‐Brain Barrier Through p65‐VEGFA‐TJs to Improve the Cognitive Function of MCAO/R Convalescent Rats,” Molecular Neurobiology 62, no. 2 (February 2025): 1451–1466, 10.1007/s12035-024-04337-8. 38995444 PMC11772513

[46] Y. Gong , Z. Yu , Y. Wang , et al., “Effect of Moxibustion on HIF‐1α and VEGF Levels in Patients With Rheumatoid Arthritis,” Pain Research & Management 2019 (2019): 4705247, 10.1155/2019/4705247. 31885755 PMC6900949

[47] E. L. Newport , A. R. Pedrosa , A. Njegic , K. M. Hodivala‐Dilke , and J. M. Muñoz‐Félix , “Improved Immunotherapy Efficacy by Vascular Modulation,” Cancers 13, no. 20 (October 2021): 5207, 10.3390/cancers13205207. 34680355 PMC8533721

[48] A. J. Vickers , E. A. Vertosick , G. Lewith , et al., “Acupuncture for Chronic Pain: Update of an Individual Patient Data Meta‐Analysis,” Journal of Pain 19, no. 5 (May 2018): 455–474, 10.1016/j.jpain.2017.11.005. 29198932 PMC5927830

[49] Y. Hasin , M. Seldin , and A. Lusis , “Multi‐Omics Approaches to Disease,” Genome Biology 18, no. 1 (May 2017): 83, 10.1186/s13059-017-1215-1. 28476144 PMC5418815

[50] K. J. Karczewski and M. P. Snyder , “Integrative Omics for Health and Disease,” Nature Reviews Genetics 19, no. 5 (May 2018): 299–310, 10.1038/nrg.2018.4. PMC5990367 29479082

[51] X. Wen , Y. Wang , C. Su , et al., “Integrating Multi‐Omics Technologies With Traditional Chinese Medicine to Enhance Cancer Research and Treatment,” QJM: An International Journal of Medicine 118, no. 11 (2025): 805–815, 10.1093/qjmed/hcaf103. 40300104

[52] K. M. Piell , C. C. Poulton , C. G. Stanley , D. J. Schultz , and C. M. Klinge , “Integrated Metabolomics and Transcriptomics Analysis of Anacardic Acid Inhibition of Breast Cancer Cell Viability,” International Journal of Molecular Sciences 25, no. 13 (2024): 7044, 10.3390/ijms25137044. 39000156 PMC11241071

[53] E. A. Eisenhauer , P. Therasse , J. Bogaerts , et al., “New Response Evaluation Criteria in Solid Tumours: Revised RECIST Guideline (Version 1.1),” European Journal of Cancer 45, no. 2 (2009): 228–247, 10.1016/j.ejca.2008.10.026. 19097774

[54] C. Wan , S. You , P. Quan , et al., “Development and Validation of the Quality‐Of‐Life Assessment System for Lung Cancer Based on Traditional Chinese Medicine,” Evidence‐Based Complementary and Alternative Medicine: eCAM 2012 (2012): 945910, 10.1155/2012/945910. 23304229 PMC3530826

[55] P. Quan , P. Y. Zheng , S. F. You , et al., “Clinical and Psychometric Validation of the Quality of Life Assessment System for Advanced Gastric Cancer Based on Traditional Chinese Medicine,” Chinese Journal of Integrative Medicine 22, no. 8 (August 2016): 581–588, 10.1007/s11655-016-2465-6. 27299458

[56] Y. Dong , L. Liu , X. Zhang , et al., “A Cross‐Sectional Study on the Application of Patient‐Reported Outcome Measurements in Clinical Trials of Traditional Chinese Medicine in Mainland China,” Frontiers in Pharmacology 14 (2023): 1159906, 10.3389/fphar.2023.1159906. 37251323 PMC10213936

[57] P. Yu , H. Wei , K. Li , et al., “The Traditional Chinese Medicine Monomer Ailanthone Improves the Therapeutic Efficacy of Anti‐PD‐L1 in Melanoma Cells by Targeting c‐Jun,” Journal of Experimental & Clinical Cancer Research 41, no. 1 (2022): 346, 10.1186/s13046-022-02559-z. 36522774 PMC9753288

